# Comparing Full and Pre-Term Neonates’ Heart Rate Variability in Rest Condition and during Spontaneous Interactions with Their Parents at Home

**DOI:** 10.3390/healthcare11050672

**Published:** 2023-02-24

**Authors:** Theano Kokkinaki, Maria Markodimitraki, Giorgos Giannakakis, Ioannis Anastasiou, Eleftheria Hatzidaki

**Affiliations:** 1 Child Development and Education Unit, Laboratory of Applied Psychology, Department of Psychology, University of Crete, 74150 Rethymnon, Greece; 2 Department of Preschool Education, University of Crete, 74150 Rethymnon, Greece; 3 Institute of Computer Science, Foundation for Research and Technology, 70013 Heraklion, Greece; 4 Cardiology Department, University Hospital of Heraklion, University of Crete, 71500 Heraklion, Greece; 5 Department of Neonatology/Neonatal Intensive Care Unit, University Hospital of Heraklion, School of Medicine, University of Crete, 71500 Heraklion, Greece

**Keywords:** heart rate variability (HRV), time-domain indices, frequency-domain indices, non-linear measurements, very low-frequency band (VLF), PNN50, preterm neonates, full-term neonates, spontaneous neonate-parent interaction

## Abstract

Background: Preterm neonates show decreased HRV compared to those at full-term. We compared HRV metrics between preterm and full-term neonates in transfer periods from neonate rest state to neonate–parent interaction, and vice versa. Methods: Short-term recordings of the HRV parameters (time and frequency-domain indices and non-linear measurements) of 28 premature healthy neonates were compared with the metrics of 18 full-term neonates. HRV recordings were performed at home at term-equivalent age and HRV metrics were compared between the following transfer periods: from first rest state of the neonate (TI1) to a period in which the neonate interacted with the first parent (TI2), from TI2 to a second neonate rest state (TI3), and from TI3 to a period of neonate interaction with the second parent (TI4). Results: For the whole HRV recording period, PNN50, NN50 and HF (%) was lower for preterm neonates compared to full-terms. These findings support the reduced parasympathetic activity of preterm compared to full-term neonates. The results of comparisons between transfer period simply a common coactivation of SNS and PNS systems for both full and pre-term neonates. Conclusions: Spontaneous interaction with the parent may reinforce both full and pre-term neonates’ ANS maturation.

## 1. Introduction

Heart rate variability (HRV) constitutes a non-invasive biomarker and refers to the physiological fluctuations and time intervals between spontaneous and successive heartbeats [1,2,3,4]. HRV is widely used to efficiently assess the regulatory activity of the autonomic nervous system (ANS) by its sympathetic and parasympathetic components and makes it possible to evaluate the balance between these two branches within the ANS [5,6]. HRV analysis has diagnostic and prognostic potential for detecting and monitoring dysregulation due to disease in neonates, and gives paramount hints about the newborns’ wellbeing and socioemotional and cognitive development [6,7,8,9].

The ANS undergoes significant maturation between 31- and 38-weeks’ gestation [10]. The sympathetic system develops early in pregnancy, while parasympathetic control emerges later in the perinatal period [4]. Gestational (GA) and postmenstrual age (PMA) have the largest influence on HRV [4,6]. The ANS of preterm infants is underdeveloped and the multiple control loops responsible for homeostasis may not yet work synergistically [6]. Prematurity delays maturation of HRV [2] (Fyfe et al., 2014) and preterm birth has been associated with decreased HRV [5] (Aye et al., 2018). Lower HRV values indicate abnormal adaptation with impaired function of the ANS and vulnerability to stress, while an increase in HRV represents physical and mental adaptability along with efficient autonomic mechanisms [9,11]. Furthermore, environmental challenges in the postnatal days play a crucial role in the development of the parasympathetic system and the maturational course of sympathetic regulation may be altered by physiological challenges in the NICU [12]. Preterm infants in the Neonatal Intensive Care Unit (NICU) experience chronic exposure to stressors [13]. When the underdevelopment of the ANS of preterm infants and NICU stressors are combined, ANS maturation of preterm neonates may be further delayed and impaired, with consequences on their overall development that persist later in life [1]. Interventions are needed to reduce the adverse environmental impacts on ANS development to mitigate exposure to stressors in NICUs and to enhance maturation of the ANS of preterm neonates [1,13].

### 1.1. HRV Variations between Full- and Pre-Term Neonates/Infants

Evidence based on measures of neonates, mainly in the NICU or in a laboratory setting, shows that HRV of preterm infants is less complex and slower compared to full-term neonates at the same postmenstrual age [11,14,15]. At birth and within the first weeks of life, preterm infants display lower scores in certain time-and frequency-domain parameters of HRV: in mean RR, lower values of root mean square of the difference between adjacent NN intervals (RMSSD), standard deviation of the NN intervals (SDNN), total power (TP) and very low (VLF) frequency power. The most significant differences have been found in the high frequency power parameter (HF), which increases with gestational age. Preterm neonates have higher or lower low frequency power values (LF) compared to full-terms [4,5,8,15,16,17,18]. As for the relative changes (%), the power in the HF and LF spectrum revealed the most marked increase with gestational age [4]. Evidence on the LF/HF ratio is contradictory. There is a negative correlation with gestational age at birth and the LF/HF ratio was higher in preterm infants [2,5,8,16], or the LF/HF ratio does not differ significantly between preterm and full-term infants [17]. Furthermore, decreased complexity of HRV dynamics in preterm compared to full-term infants is evidenced by non-linear indices, as this has been shown by only three relevant studies. Compared to full-terms, preterm infants have more linear and less chaotic patterns, smaller values of sample entropy, higher values of α1 but no variations in α2 [8,15,18].HRV variations between preterm and full-term infants are evident right after birth; they remain at preterm theoretical term age and they persist even beyond term-equivalent age [2,8]. Methodological variations and the lack of consensus in neonatal HRV analysis makes synthesis and comparisons between investigations very difficult, if not impossible [3]. However, the above review provides evidence that, generally, premature infants show decreased HRV compared to full-terms according to differences in time and frequency domain parameters and in non-linear indices. These variations imply that early in life, compared to the HRV of term counterparts, HRV of preterm infants is characterized by a reduction in sympathetic, and even more markedly, parasympathetic activities, and a relative sympathovagal imbalance, which results in the impaired function of ANS [2,4,5,15,16].

### 1.2. HRV of Full- and Pre-Term Infants in Different Contexts and Conditions and in Interactions with Their Parents

Evidence of HRV variations in preterm and full-term infants in different conditions/contexts, involving mostly mother–infant sensory stimulation, is rarely investigated in the naturalistic environment, therefore studies are limited and the evidence is contradictory. In particular, in preterm infants(aged 33 weeks), the LF/HF ratio was similar during caregiving epochs and sleep epochs, though the LF/HF ratio increased during periods of caregiving for massage-treated male infants. This suggests an increase in sympathetic response during a physiologically demanding time period [13]. In the first 4post-term months, HF of preterm infants was higher during pre-feeding, decreased during feeding and returned to the pre-feeding level during post-feeding, though LF did not show a similarly consistent pattern. This shows an adaptive response to stimulation that requires increased attention, or metabolic output [19]. Furthermore, transfer of the preterm infant (34 weeks) from the open-crib to Kangaroo Care (KC, sensory stimulation from being in skin-to-skin contact with the mother’s chest) decreased the values of LF and HF and, conversely, the LF/HF ratio was higher in KC. Overall, KC produced changes in HRV that indicated a decrease in stress [10]. In the course of maternal natural breathing and physical contact, the LF power in 3–5-month-old full-term infants did not differ according to the period (pre-rest, respiration, post-rest). No correlation was found between the mothers’ LF power and 3–5-month-old infants’ LF power during the paced breathing period. Young infants showed a delayed increase in the LF components after termination of maternal-paced breathing, possibly due to their immaturity [9]. It is difficult to integrate these results due to the methodological heterogeneity of relevant studies. Despite that, this literature provides evidence of HRV variations in pre-term and full-term neonates according to various contexts of sensory stimulation.

### 1.3. Research Questions

In this study, we addressed the following research questions:

1. Do HRV parameters (time-domain, frequency-domain indices and non-linear measurements) of preterm and full-term infants vary in the total duration of three transfer periods from rest state to spontaneous interaction with the parent, and vice versa?
2. Do HRV parameters of preterm and full-term infants vary between three transfer periods, that is between: (a) rest state 1 (TI1) and spontaneous interaction between neonate-parent 1 (mother or father) (TI2), (b) spontaneous interaction between neonate-parent 1 (TI2) and rest state 2 (TI3), and (c) rest state 2 (TI3) and spontaneous interaction between neonate-parent 2 (mother or father) (TI4)?

## 2. Material and Methods

### 2.1. Participants

One hundred and two mothers, fathers and neonates participated in the study in two groups. *The first group* included 18 parents and their infants born at full-term ≥ 37 weeks gestational age (GA) with no medical complications. *The second group* consisted of 28 parents and their preterm infants. Ninety three percent of preterm infants included in this study were moderate-to-late pre-terms (32–36 weeks GA) and only 7% of them were healthy pre-terms with a GA of 31 weeks. Exclusion criteria included: perinatal asphyxia, neurological pathologies, malformation syndromes and major malformations, sensory deficits, metabolic genetic disease, or CNS infection.

Six mothers in the full-term group and three mothers in the preterm group were not included in the final sample due to: delayed answer to the researcher for participation in the study, neonates’ hospitalization, or time constraints. No differences were observed between participating and non-participating families on family demographic, or infant medical status. Demographics and infant medical status of the two groups are reported in Table 1 and Table 2. The data show no group differences in maternal and paternal education years. Mothers and fathers of premature neonates were slightly older than parents of full-terms. All families were middle-class [20], both parents were older than 20 years of age, they did not suffer from a psychiatric illness, and they did not have issues with drug or substance abuse; mothers were married to the child’s father and in all families at least one parent was employed.

### 2.2. Procedure

After ethical approval (see in notes), parents were approached shortly after birth at the Neonatal Intensive Care Unit (NICU) of the Neonatology Clinic of the General University Hospital of Crete (Greece) (preterm) and at private Maternity/Gynecological Clinic Mitera of Heraklion (full-term). Firstly, the medical staff of the above clinics asked the parent’s consent to provide their communication information to the members of the research team. After parental consent, a neonatologist and a psychologist (both members of the research team) informed the parents about the aim and the procedure of the study. Parents who accepted to participate were asked to sign the consent form. In the course of the same meeting, parents were asked to answer questions regarding family sociodemographic characteristics and the neonate’s birth characteristics. Then, the first visit to the family’s home for the video-recording was scheduled at a time when both parents would be available and when the neonate was expected to be alert.

The video-recording was performed within the first four to five weeks after birth at term-equivalent age for both groups, that is, for preterm neonates at mean PMA 39.57 weeks (SD = 2.41, min–max = 37–45 weeks) and for full-term neonates at mean PMA 42.55 (SD = 1.75, min–max = 39–46 weeks). Newborns with a post-conceptual age of more than 38 weeks are relatively mature in terms of sympathovagal balance (Javorka et al., 2017).The whole recording lasted 30 min and it was segmented in three time intervals (TI) as follows TI1: resting state 1 (no neonate–parent interaction, HRV measurement of the infant in a supine position) (7 min), TI 2: interaction of the neonate with the first parent (8 min), TI3: resting state 2 (no neonate–parent interaction, HRV measurement of the infant in a supine position) (7 min), TI4: interaction of the neonate with the second parent (8 min). For the interaction, the only instruction given to the parents was: “Play as you normally do with your young baby”. The recording took place in a room and at a position chosen by the parents prohibiting any third-party intervention. If the neonate became distressed, or either the parents, or the researcher considered that the visit should be postponed for some reason, it was rescheduled as soon as possible thereafter.

### 2.3. Heart Rate Variability Analysis

#### 2.3.1. Heart Rate Variability Data Collection

Neonate HRV measurements were carried out through SEER 1000, ECG Recorder, and General Electric (Version 1.0, 2067634-077 Revision F). The device was used by a trained operator under the direct supervision of a licensed healthcare practitioner. The device is suitable for use for pediatric patients, including those patients weighing less than 10 kg. For the data collection, the device was connected via Bluetooth to an Android mobile smartphone. Recording and HRV measurement was stopped if there was excessive restlessness or crying.

#### 2.3.2. Heart Rate Variability Data Processing

Once the recording was completed, an ECG analysis software (General Electric, Athens, Greece)package was used for data collection. The ECG preprocessing and the HRV parameters extraction analysis was performed using custom scripts written on the MATLAB r2018b platform. During the preprocessing phase, the ECG signal was detrended by subtracting time series polynomial fit or order 60. The R components of the QRS complex were detected and the RR Intervals (RRI) were calculated. The ectopic heartbeats (irregular heartbeats deviated from normal) were also detected and excluded by adopting the HRV signal approach (percentage change of 70% over the averaged previous 5 heartbeats). The whole preprocessing procedure is described in [21].

#### 2.3.3. Heart Rate Variability Analysis

Short-term recordings of HRV parameters of premature and full-term neonates were performed [22,23]. Calculated HRV features were based on time-domain indices (quantification of the amount of HRV observed during monitoring periods), frequency-domain values (calculation of the absolute or relative amount of signal energy within component bands) and non-linear measurements (quantification of the unpredictability and complexity of a series of interbit intervals) [14,18,22] (Table 3).

### 2.4. Statistical Analysis

Data were tested for their normality using the Kolmogorov–Smirnov test. Firstly, they were analyzed, controlling for differences in their HRV parameters between the two groups (full-term and preterm infants) for the whole recording time and for each time interval separately, using the independent samples *t*-test or Mann–Whitney test. Secondly, the effect of parent interaction was investigated, controlling for pairwise differences between two conditions (no interaction, parent interaction) within each group (full-term and preterm infants) using Pairwise *t*-test or Wilcoxon signed-rank test. The statistical significance level was set to a = 0.05. All statistical analyses were performed using custom scripts in the MATLAB R2018b platform environment.

It should be noted that we compared the NN50 between the two groups only in the total duration of the recording. The NN50 was excluded from the analysis between rest states 1 and 2 and interaction with the first/second parent as these had different durations. Only the pNN50 was utilized in these cases as it is not affected by the recording duration.

## 3. Results

### 3.1. Comparing HRV Parameters between Full- and Pre-Term Neonates in the Whole Recording Duration

The comparison of HRV parameters between full- and preterm infants for the whole recording duration showed that RMSSD (*U* = 344, *z* = 2.060, *p* = 0.039), the pNN50 (*U* = 344, *z* = 2.059, *p* = 0.037), the HF (%) (*U* = 341, *z* = 1.992, *p* = 0.046) and the VLF (%) (*t*(44) = 0.424, *p* = 0.046) of preterm neonates was statistically significantly reduced in relation to the full-terms (Table 4).

### 3.2. Comparing HRV Parameters of Full- and Pre-Term Neonates between Resting Condition 1 and Interaction with the First Parent

The comparison of HRV parameters of full-term infants between resting condition 1 and interaction with the first parent (Table 5) shows that HR_m_(*t*(17) = −3.61, *p =* 0.002) and total power (*z* = −2.11, *p =* 0.035) was increased, while the VLF peak (*z* = 2.22, *p =* 0.026), DFA α (*t*(17) = 4.07, *p* = 0.001), DFA α_1_(*t*(17) = 2.44, *p* = 0.030), DFA α_2_ (*t*(17) = 3.48, *p* = 0.004) were significantly reduced.

The comparison of HRV parameters of preterm infants between resting condition 1 and interaction with the first parent (Table 6) shows that the preterm HR_m_(*t*(27) = −3.45, *p =* 0.002), total power (*t*(27) = −2.64, *p* = 0.014) and VLF (%) (*t*(27) = −4.87, *p <* 0.001) was increased, while the DFA α2 (*t*(27) = 2.49, *p* = 0.021)was reduced.

### 3.3. Comparing HRV Parameters of Full- and Pre-Term Infants between Interaction with the First Parent and Resting Condition 2

For the interaction between the first parent and resting condition 2, preterm HR_m_(*t*(27) = 2.78, *p =* 0.010), total power (*z* =2.02, *p =* 0.043), and VLF (%) (*t*(27) = 3.35, *p =* 0.002) decreased, while LF (%) (*t*(27) = −2.366, *p =* 0.025) increased (Table 7).

For the interaction between the first parent and resting condition 2, full-term infants’ VLF and DFAa increased (Table 8).

### 3.4. Comparing HRV Parameters between Full- and Pre-Term Infants between Resting Condition 2 and Interaction with the Second Parent

For the interaction between rest condition 2 and the second parent, VLF % increased (Table 9 and Table 10). However, LF% decreased only for full-terms and VLF peak decreased only for pre-terms.

**Figure 2:**
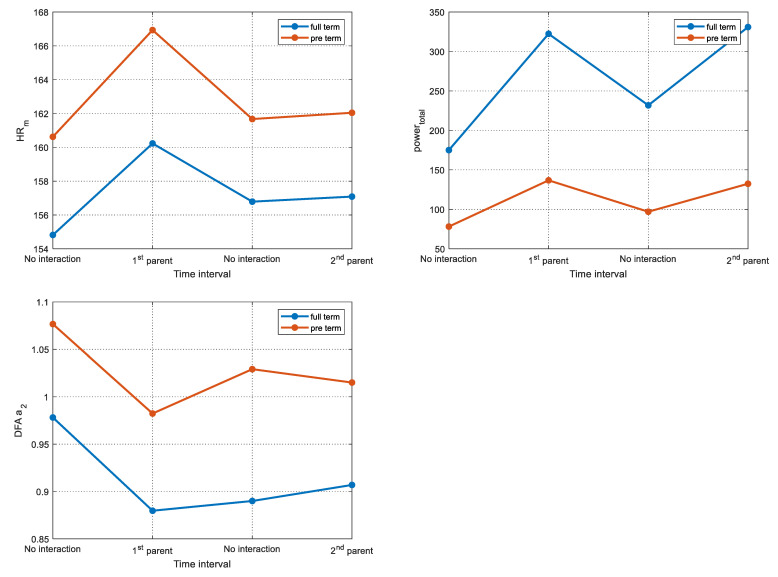
HRV parameters (HR_m_, total power, DFA α_2_) behavior in the investigated interaction patterns (resting condition 1 (no interaction), interaction with the parent, resting condition 2 (no interaction), interaction with the parent).

The significant HRV behavior parameters in the investigated interaction patterns (resting condition 1 (no interaction), interaction with the 1st parent, resting condition 2 (no interaction), interaction with the 2nd parent) are depicted in Figure 2.

## 4. Discussion

We aimed to compare HRV parameters between full-term and preterm neonates, and between transfer periods from rest state to spontaneous interaction of neonates with their parents at home, and vice versa.

A comparison of HRV parameters between full- and preterm infants in the four time intervals, in total, showed that PNN50, NN50and HF (%) of preterm infants was significantly decreased compared to full-terms. This is consistent with findings showing that preterm infants score lower in time-domain parameters compared to full term infants, and with evidence showing that increasing prematurity has been associated with lower HF [5,12,15,16]. The pNN50 is closely correlated with PNS activity and the HF band reflects parasympathetic activity [14,22]. Reduced pNN50 and HF(%) of premature infants compared to full-terms may be attributed to the early disruption of autonomic development, which causes immaturity of ANS [5,24]. The sympathetic system shows steady development throughout the fetal period and develops earlier than the parasympathetic system. The latter begins to develop during the first trimester and development continues until birth but it undergoes accelerated maturation at 25–32 weeks’ gestation. The normal steep increase in vagal tone (which reflects the parasympathetic division activation) occurs around 37–38 weeks at a time when premature newborns may already have been in an ex utero environment. In infants born prematurely, the normal third trimester increase in parasympathetic tone may be dampened in the ex utero environment, compared to that of the inutero third trimester fetus [2,14,18,24]. Stressful environmental stimuli in the NICU (e.g., invasive procedures, mechanical ventilation, loud noise, and bright lights) may have impeded normal maturation of the ANS [14,18]. Deficits in HRV parameters in the preterm population may persist after birth up to term-equivalent age [2,5,8].

We indicated that between TI1 and TI2, certain common HRV parameters changed in the same direction between full- and pre-terms, while others varied. In particular, HR and total power increased and *a2* decreased for both groups, while DFA, DFA *a1* and VLF peak decreased for full terms, and VLF (%) increased for pre-terms. HR increase indicates a rise in SNS activity [6,25]. Total power, the sum of the energy of VLF, LF, and HF bands for short-terms recordings, represents the overall variability [26,27]. HF are expressions of PNS activation, while LF contains contributions of both the SNS and PNS influences [22]. Thus, a total power increase implies coactivation of the SNS and PNS systems. Non-linear indices reflect the unpredictability of a time series, which results from the complexity of the mechanisms that regulate HRV [22]. These measurements quantify the properties of heart rate dynamics, such as response patterns and self-correlation, which are caused by complex interplays between vagal and sympathetic regulations [28]. In this context, *α2* characterizes ultraslow changes in the heart rate (below the frequency of sympathetic tone) and reflects the regulatory mechanisms that limit fluctuation of the beat cycle [22]. It is noted that a decrease in DFAa reflects an adverse adaptive situation not related to “slow recovery” processes or vagal activity (URL: mathnet.ru/php/archive.phtml?wshow=paper&jrnid=ivp&paperid=200&option_lang=eng accessed on 17 February 2023) Taken together, for both full and pre-term infants, the transfer period from TI1 to TI2 is associated with an increase in overall variability and coactivation of the SNS and PNS systems, along with a decrease in the regularity of heartbeats.

An interesting finding of this study is that between TI1-TI2 and TI2-TI3, the HR of pre-terms changed but in opposite directions. In particular, in the transfer period, TI1-TI2, preterm HR increased, while in TI2-TI3, preterm HR decreased. Thus, between transfer periods TI1-TI2-TI3, we indicated a fluctuating SNS activation of preterm infants. Between TI3-TI4, LF (%) decreased only for full-terms, implying decreases in SNS and PNS influences. Given that an infant’s state influences arousal, attention and affect [29], these patterns of SNS and PNS activation in TI3-TI4 may be attributed to fatigue of young infants after 22-min transfer periods [7 min (rest state 1) + 8 min (interaction) + 7 min (rest state 2)].

## 5. Conclusions

In accordance with the previous literature, a comparison of HRV parameters across the four time intervals showed lower scores in certain time-domain parameters (PNN50, NN50) and in HF (%), a frequency-domain parameter of preterm infants compared to full-term infants. These findings support the reduced parasympathetic activity of preterm compared to full-term neonates. Furthermore, HRV metric changes across the transfer periods from rest conditions to spontaneous neonate-parent interaction, and vice versa, imply a common coactivation of the SNS and PNS systems for both full and pre-term neonates (TI1-TI2), a fluctuating SNS activation for pre-terms (TI1-TI2 and TI2-TI3), and decreases in SNS and PNS activation for full-terms (TI3-TI4).

### 5.1. Limitations of This Study

To deepen our understanding of HRV variations between preterm and full-term infants, larger samples are needed for the measurement of both short- and long-term HRV metrics. An investigation into the correlation of HRV parameters with maternal lifestyle and delivery mode is needed [30,31,32].Due to the small sample size of preterm infants, we were not powered to detect differences between subgroups according to gestational age and we did not control variations in HRV parameters between full- and preterm infants according to parent gender. This is important because mothers and fathers vary in the interactive patterns with their infants [33] and between full-term and preterm infants [34].

### 5.2. Implications for Practice

The findings of this study highlight the utility of HRV in neonatology and the importance of introducing the HRV in as many NICUs as possible in order to improve neonatal care [14]. In order to enhance family-centered and family-integrated developmental care practices in the NICU, high priority should be given to facilitate and reinforce the parent–preterm infant physical and emotional closeness and parental involvement in the infant’s care. In this context, the concept of parents as “partners in care” rather than “visitors” should be further supported. This will have short- and long-term positive implications for infant development, benefits for parental mental health and for the development of parent–infant bonding, along with implications for the wellbeing of health professionals. Furthermore, it is vital to increase the awareness of healthcare specialists about the critical need to enable parents’ access to the NICU and an active engagement of parents in the primary care of hospitalized newborns [35,36,37].

## Figures and Tables

**Figure 1:**
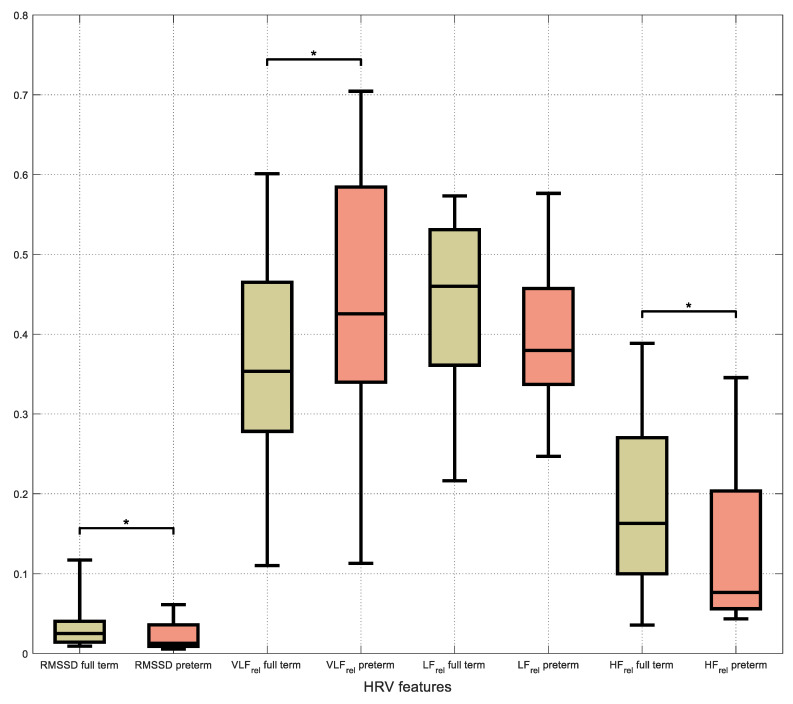
Box-plots of the HRV parameters RMSSD, VLF, LF, HF for the whole recording duration. Asterisk denotes statistically significant difference at 0.05 level.

**Table 1:** Neonate medical variables of the two groups of the sample.

	Neonate Medical Variables					
	Preterm Neonates (N = 28)			Full-Term Neonates (N = 18)		
	M	SD	range	M	SD	range
GA * (weeks)	34.03	1.52	31–36	38.61	1.09	37–40
PMA ** (weeks) at video-recording	39.57	2.41	37–45	42.55	1.75	39–46
Birth weight ***	2200	450.68	1520–3240	3310	208.12	2820–3750
Birth height	45.16	2.47	41–50	51.05	1.06	49–53
Male/Female ratio	19/9			10/8		

Notes: * GA: Gestational age; ** PMA: Postmenstrual age; *** 23 neonates(82.1% of pre-terms) had birth weight < 2500 g and 8 neonates (28.5%) <2000 g.

**Table 2:** Family demographic of the two groups of the sample.

	Parental Characteristics					
	Families of Preterm Neonates (N = 28)			Families of Full-Term Neonates (N = 18)		
	M	SD	range	M	SD	range
Maternal age (years)	35.71	5.28	25–49	32.88	5.00	24–42
Maternal education (years)	15.28	2.50	6–18	15.88	2.32	12–18
Paternal age (years)	41.64	5.77	31–56	37.05	5.99	29–47
Paternal education (years)	14.78	2.79	6–18	15.00	2.49	12–18

**Table 3:** HRV parameters measured in full- and pre-term infants.

Parameter	Definition	Unit
**Time-domain**		
HR_m_	Mean heart rate	bpm
HR_std_	Standard deviation of instantaneous heart rate values	bpm
HRV triangular index	Integral of the density of the RR interval histogram divided by its height	-
SDNN	Standard deviation of NN intervals	s
rMSSD	Root mean square of consecutive RR interval differences	s
NN50	Number of adjacent NN intervals that differ from each other by more than 50 ms	-
pNN50	Percentage of successive NN intervals that differ by more than 50 ms	%
**Frequency-domain**		
VLF_peak	Peak frequency of the very low-frequency band	Hz
LF_peak	Peak frequency of the low-frequency band	Hz
HF_peak	Peak frequency of the high-frequency band	Hz
VLF (%)	Normalized VLF power	-
LF (%)	Normalized LF power	-
HF (%)	Normalized HF power	-
LF/HF	Ratio of LF-to-HF power	-
Total power	Total power of the ECG spectrogram	Hz
**Non-linear**		
DFA α	Detrended fluctuation analysis coefficient	-
DFA α_1_	Detrended fluctuation analysis, which describes short-term fluctuations	-
DFA α_2_	Detrended fluctuation analysis, which describes long-term fluctuations	-

**Table 4:** Comparison of HRV parameters between full- and preterm infants for the whole recording duration.

HRV Features	Full-Term Neonates	Preterm Neonates	*p*-Value	Difference
HR_m_	157.5	162.9	0.134	ns
SDNN	0.042	0.035	0.156	ns
HR_std	18.9	16.2	0.359	ns
**RMSSD**	**0.037**	**0.022**	**0.039**	**↓**
**NN50**	**271.1**	**99.0**	**0.039**	**↓**
**pNN50**	**6.4**	**2.3**	**0.037**	**↓**
HRV_Tri	8.6	8.0	0.465	ns
VLF_peak	0.020	0.019	0.674	ns
LF_peak	0.06	0.06	0.767	ns
HF_peak	0.18	0.18	0.743	ns
Total power	367.6	233.1	0.209	ns
**VLF (%)**	**0.359**	**0.448**	**0.046**	**↑**
LF (%)	0.43	0.40	0.290	ns
**HF (%)**	**0.19**	**0.13**	**0.046**	**↓**
LF/HF	3.59	4.87	0.180	ns
DFA α	1.03	1.04	0.923	ns
DFA α_1_	1.10	1.10	0.989	ns
DFA α_2_	0.96	0.95	0.869	ns

Note: Bold type denotes a statistically significant difference between groups. The differentiations are depicted in Figure 1. Ns means ’non-significant’ and arrows show the direction of variation for a specific parameter.

**Table 5:** Comparison of full-term infants’ HRV parameters between resting condition 1 and interaction with the 1st parent.

HRV Feature	Resting Condition 1	Interaction between Full-Term Neonate-1st Parent	*p*-Value	Difference
HR_m_	154.8	160.2	**0.002**	**↑**
SDNN	0.033	0.038	0.344	ns
HR_std	14.9	16.8	0.247	ns
RMSSD	0.029	0.039	0.112	ns
pNN50	4.6	7.8	0.085	ns
HRV_Tri	6.7	6.9	0.654	ns
**VLF_peak**	**0.037**	**0.035**	**0.026**	**↓**
LF_peak	0.07	0.06	0.144	ns
HF_peak	0.20	0.19	0.794	ns
**Total power**	**175.0**	**322.4**	**0.035**	**↑**
VLF (%)	0.033	0.064	0.171	ns
LF (%)	0.65	0.61	0.165	ns
HF (%)	0.28	0.28	0.947	ns
LF/HF	3.47	3.23	0.665	ns
DFA α	1.02	0.95	0.059	ns
DFA α_1_	1.01	0.96	0.405	ns
**DFA α_2_**	**1.03**	**0.91**	**0.004**	**↓**

Note: Bold type denotes a statistically significant difference between groups. Ns means ’non-significant’ and arrows show the direction of variation for a specific parameter.

**Table 6:** Comparison of preterm infants’ HRV parameters between resting condition 1 and interaction with the 1st parent interaction and interaction with the 1st parent.

HRV Feature	Resting Condition 1	Interaction between Preterm Neonate-1st Parent	*p*-Value	Difference
HR_m_	**160.6**	**166.9**	**0.002**	**↑**
SDNN	0.030	0.032	0.511	ns
HR_std	14.1	16.1	0.125	ns
RMSSD	0.018	0.025	0.064	ns
pNN50	1.7	3.2	0.059	ns
HRV_Tri	6.6	6.4	0.650	ns
VLF_peak	0.036	0.036	0.289	ns
LF_peak	0.07	0.06	0.168	ns
HF_peak	0.18	0.19	0.367	ns
**Total power**	**78.1**	**136.7**	**0.014**	**↑**
**VLF (%)**	**0.043**	**0.102**	**0.000**	**↑**
LF (%)	0.68	0.63	0.122	ns
HF (%)	0.25	0.23	0.653	ns
LF/HF	4.13	4.01	0.856	ns
DFA α	1.11	1.06	0.117	ns
DFA α_1_	1.10	1.11	0.860	ns
**DFA α_2_**	**1.07**	**0.98**	**0.021**	**↓**

Note: Bold type denotes a statistically significant difference between groups. Ns means ’non-significant’ and arrows show the direction of variation for a specific parameter.

**Table 7:** Comparison of preterm infants’ HRV parameters between interaction with the 1st parent and resting condition 2.

HRV Feature	Interaction between Preterm Infant-1st Parent	Resting Condition 2	*p*-Value	Difference
HR_m_	**166.9**	**161.7**	**0.010**	**↓**
SDNN	0.032	0.032	0.983	ns
HR_std	16.1	14.8	0.361	ns
RMSSD	0.025	0.019	0.255	ns
pNN50	3.2	1.9	0.211	ns
HRV_Tri	6.4	6.4	0.958	ns
VLF_peak	0.036	0.036	0.112	ns
LF_peak	0.06	0.07	0.062	ns
HF_peak	0.19	0.21	0.608	ns
**Total power**	**136.7**	**96.9**	**0.043**	**↓**
**VLF (%)**	**0.102**	**0.046**	**0.002**	**↓**
**LF (%)**	**0.63**	**0.70**	**0.025**	**↑**
HF (%)	0.23	0.23	0.848	ns
LF/HF	4.01	4.30	0.647	ns
DFA α	1.06	1.09	0.183	ns
DFA α_1_	1.11	1.23	0.121	ns
DFA α_2_	0.98	0.99	0.833	ns

Note: Bold type denotes a statistically significant difference between groups. Ns means ’non-significant’ and arrows show the direction of variation for a specific parameter.

**Table 8:** Comparison of full-term infants’ HRV parameters between interaction with the 1st parent and resting condition 2.

HRV Feature	Interaction between Full-Term Neonate-1st parent	Resting Condition 2	*p*-Value	Difference
HR_m_	160.2	156.8	0.142	ns
SDNN	0.038	0.039	0.988	ns
HR_std	16.8	16.4	0.948	ns
RMSSD	0.039	0.031	0.097	ns
pNN50	7.8	6.5	0.193	ns
HRV_Tri	6.9	7.2	0.462	ns
VLF_peak	0.035	0.037	**0.022**	**↑**
LF_peak	0.06	0.06	0.580	ns
HF_peak	0.19	0.18	0.862	ns
Total power	322.4	231.8	0.145	ns
VLF (%)	0.064	0.039	0.256	ns
LF (%)	0.61	0.67	0.145	ns
HF (%)	0.28	0.25	0.239	ns
LF/HF	3.23	4.20	0.248	ns
**DFA α**	0.99	1.11	**0.019**	**↑**
DFA α_1_	1.02	1.22	0.060	ns
DFA α_2_	0.91	1.00	0.118	ns

Note: Bold type denotes a statistically significant difference between groups. Ns means ’non-significant’ and arrows show the direction of variation for a specific parameter.

**Table 9:** Comparison of full-term infants’ HRV parameters between resting condition 2 and interaction with the 2nd parent.

HRV Feature	Resting Condition 2	Interaction between Full-Term Neonate-2nd Parent	*p*-Value	Difference
HR_m_	156.8	157.1	0.882	ns
SDNN	0.039	0.041	0.487	ns
HR_std	16.4	18.7	0.078	ns
RMSSD	0.031	0.038	0.147	ns
pNN50	6.5	6.2	0.492	ns
HRV_Tri	7.2	6.8	0.366	ns
VLF_peak	0.037	0.034	0.126	ns
LF_peak	0.06	0.06	0.673	ns
HF_peak	0.18	0.18	0.812	ns
Total power	231.8	330.9	0.170	ns
**VLF (%)**	0.039	0.119	**0.013**	**↑**
**LF (%)**	0.67	0.59	**0.045**	**↓**
HF (%)	0.25	0.25	0.994	ns
LF/HF	4.20	3.25	0.293	ns
DFA α	1.11	1.06	0.223	ns
DFA α_1_	1.22	1.15	0.451	ns
DFA α_2_	1.00	0.98	0.548	ns

Note: Bold type denotes a statistically significant difference between groups. Ns means ’non-significant’ and arrows show the direction of variation for a specific parameter.

**Table 10:** Comparison of preterm infants’ HRV parameters between resting condition 2 and interaction with the 2nd parent.

HRV Feature	Resting Condition 2	Interaction between Preterm Neonate-2nd Parent	*p*-Value	Difference
HR_m_	161.7	162.0	0.812	ns
SDNN	0.032	0.031	0.699	ns
HR_std	14.8	13.8	0.394	ns
RMSSD	0.019	0.019	0.964	ns
NN50	20.1	22.6	0.833	ns
pNN50	1.9	1.9	0.737	ns
HRV_Tri	6.4	6.8	0.261	ns
**VLF_peak**	0.036	0.034	**0.050**	**↓**
LF_peak	0.07	0.06	0.125	ns
HF_peak	0.21	0.18	0.463	ns
Total power	96.9	132.4	0.116	ns
**VLF (%)**	0.046	0.107	**0.008**	**↑**
LF (%)	0.70	0.66	0.130	ns
HF (%)	0.23	0.20	0.394	ns
LF/HF	4.30	5.07	0.384	ns
DFA α	1.09	1.06	0.308	ns
DFA α_1_	1.23	1.13	0.091	ns
DFA α_2_	0.99	0.97	0.536	ns

Note: Bold type denotes a statistically significant difference between groups. Ns means ’non-significant’ and arrows show the direction of variation for a specific parameter.

## Data Availability

The datasets generated during and/or analyzed during the current study are available from the corresponding author on reasonable request.
